# Efficacy and Safety of Brivaracetam in Persons With Epilepsy in a Real-World Setting: A Prospective, Non-Interventional Study

**DOI:** 10.7759/cureus.50313

**Published:** 2023-12-11

**Authors:** Fowzia Siddiqui, Bashir A Soomro, Mazhar Badshah, Ehsan U Rehman, Ahsan Numan, Amer Ikram, Muhammad Wazir Ali Khan, Sajjad Ali, Husnain Hashim, Jawwad-us Salam, Asad Akram, Muhammad Irfan Hashmat, Shahid Iqbal, Muhammad Zeeshan Javed, S. Zafar Iqbal, Atif Maqsood, Assadullah Khan, Neeta Maheshwary, Muhammad Athar Khan

**Affiliations:** 1 Neurology, The Aga Khan University Hospital (AKUH), Karachi, PAK; 2 Neurology, Ziauddin University, Karachi, BOL; 3 Neurology, Pakistan Institute of Medical Sciences, Islamabad, PAK; 4 Neurology, Akhtar Saeed Medical and Dental College, Islamabad, PAK; 5 Neurology, Mayo Hospital, King Edward Medical University, Lahore, PAK; 6 Neurology, Doctors Hospital Lahore, Lahore, PAK; 7 Neurology, Sheikh Zayed Medical College, Rahim Yar Khan, PAK; 8 Neurology, Dar ul Shifa Hospital Sialkot, Sialkot, PAK; 9 Neurology, Fauji Foundation Hospital, Rawalpindi, PAK; 10 Medicine/Neurology, Dow International Medical College, Dow University of Health Sciences, Karachi, PAK; 11 Neurology, Patel Hospital, Karachi, PAK; 12 Neurology, Civil Hospital Karachi, Karachi, PAK; 13 Neurology, Rehman Medical Institute, Peshawar, PAK; 14 Neurology, Chenab General Hospital, Multan, PAK; 15 Neurology, Tahsil Head Quarter (TQH) Hospital Sadiqabad, Sadiqabad, PAK; 16 Neurology, Aziz Fatima Hospital, Faisalabad, PAK; 17 Neurology, Lady Reading Hospital, Peshawar, PAK; 18 Medical Affairs, Helix Pharma Pvt. Ltd, Karachi, PAK; 19 Community Medicine, Liaquat College of Medicine & Dentistry, Karachi, PAK

**Keywords:** efiicacy, focal seizure, antiepileptic drug, epilepsy, brivaracetam

## Abstract

Background and aim: Epilepsy stands out as one of the most prevalent neurological conditions. Brivaracetam (BRV) is a noteworthy antiseizure medication (ASM) distinguished by its pronounced and selective interaction with the synaptic vesicle protein 2A (SV2A) within the brain. Prior investigations, including regulatory trials, post-marketing assessments, and comparative meta-analyses, have consistently underscored BRV's equivalency in efficacy and superior tolerability when pitted against other antiseizure drugs. This study aimed to evaluate the effectiveness, safety, and acceptability of BRV in treating epileptic patients in the Pakistani population.

Methods: This prospective observational study, conducted in Pakistan from February to December 2022, employed a non-probability consecutive sampling technique. This study included 368 adult patients diagnosed with epilepsy, with a focus on those aged 18 and above experiencing focal seizures. Demographic data, clinical history, seizure types, and epilepsy profiles were recorded. Patients were administered BRV (Brivera; manufactured by Helix Pharma Pvt Ltd., Sindh, Pakistan) monotherapy therapy under physician guidance and followed up for three months. The study assessed changes in seizure frequency, side effects, and drug resistance at baseline, 14^th^ day, and 90^th^ day. Safety aspects were monitored, including documenting any adverse effects associated with BRV therapy.

Results: A total of 368 epileptic patients were included in this study, of which 287 (61.3%) were males and 181 (38.7%) were females. The mean age was 32.91±17.11 years. The mean number of seizures at the baseline visit was 5.74±6.21, at 14 days was 2.89±3.84 and at 90 days was 1.73±5.01 (p<0.001). Overall, a more than 50% reduction in seizure episodes was achieved in 178 (56.3%) patients at day 90, and less than 50% reduction in seizure episodes was achieved by 95 (26.8%) patients on Day 14, with a highly significant association between them (p<0.001). Among 316 patients, only 41 (4.4%) of all BRV-treated patients experienced adverse events; Of these 41 patients, 17 (41.7%) reported dizziness and 14(34.2%) reported behavioral issues.

Conclusions: Epileptic patients receiving BRV demonstrated a substantial reduction of greater than 50% seizure episodes at the end of follow-up visits. Moreover, BRV exhibited fewer adverse effects in individuals with epilepsy.

## Introduction

Epilepsy is the world's fourth most common neurologic disease [1]. There are many causes of epilepsy, mostly with underlying cerebral dysfunction [2]. Globally, according to a WHO report, approximately 50 million people are affected by epilepsy, which amounts to about 80 cases per 100,000 individuals annually [3]. Epilepsy diminishes the patient's quality of life, and research and surveys have shown that the risk of psychiatric disorders increases with epilepsy [4]. According to the International League against Epilepsy's operational classification of epilepsy, they are focal, generalized, combined, and idiopathic [4]. A Korean epidemiological study for patients with epilepsy showed that a higher incidence is accounted for by focal epilepsy (78.1%) and generalized epilepsy affects approximately 8% of adult patients [5].

Epidemiological studies on epilepsy in Pakistan are scarce, particularly in remote and challenging regions. Despite a population exceeding 188.2 million, comprehensive investigations into this neurological disorder are lacking. Previous studies have shown that Khyber Pakhtunkhwa, Pakistan, has a high incidence of epilepsy, a high proportion of individuals in a low socioeconomic background, and a high treatment gap [6]. A study conducted in educational institutions at Sheringal, Khyber Pakhtunkhwa, Pakistan, aimed to address this gap. The targeted population for this study was 5308 individuals, revealing that 2.15% (n=114) were confirmed epilepsy patients. Notably, a higher prevalence rate per thousand of the population was observed in professional settings (54.5%) compared to primary-level institutions (29.4%) [7]. Another study revealed that 1.166% (11.9/1000) of the population in Tehsil Mastu of Chitral, Pakistan, have epilepsy. The majority of epilepsy patients are male (62.5%), almost double that of females (37.5%). The majority of the patients (40.6%) belong to the age group of 10-20 years followed by 21-30 years (25 %) [8]. 

Administration of anti-seizure medicines (ASMs) or surgical procedures are the treatment possibilities for focal epilepsy depending on the cause of the epilepsy [9,10]. However, surgical intervention for epilepsy is not opted for in most cases because of the restricted indications and high rate of refusal by patients [11,12]. ASMs are the most preferred method to treat focal epilepsy, and these drugs can control almost two-thirds of the patients [3,9]. The selection of ASMs to be recommended is based on consideration of their efficacy, safety, and side effect profile along with other factors like EEG findings, past medical history, gender, and associated medicines [9]. Nonetheless, only a few researches have directly related the efficiency and safety of many ASMs that can be helpful in the selection of accurate drugs for patients with epilepsy.

Although clinical trials have recognized the effectiveness and safety of ASMs, the findings of studies are occasionally inconsistent because of the small sample size [13]. Despite the existence of numerous guidelines by the American Academy of Neurology (AAN) and the National Institute for Health and Care Excellence (NICE) to direct medical treatment for epilepsy, there is little agreement on the suggested ASMs for the management of focal epilepsy [9,14-16].

Currently, brivaracetam (BRV) is an ASM with higher affinity and selectivity for synaptic vesicle protein 2A in the brain, around 20 times greater than that of levetiracetam [17,18]. Numerous clinical trials and post-marketing studies investigated the efficacy and acceptability of BRV in the treatment of epilepsy [19-21]. In the United States, BRV is available as monotherapy and adjuvant therapy in pediatric and adult patients with focal (partial-onset) seizures [22], and it is also available in Europe as adjuvant therapy in children and adults with focal seizures with or without secondary generalization [23]. ASM monotherapy is suggested for newly diagnosed epileptic patients to control seizures while reducing the adverse consequences and preventing drug-to-drug interactions [24]. The collective data of Phase IIb/III and follow-up analysis of more than eight years confirmed the safety, acceptability, and effectiveness of adjuvant BRV administered in treatment for focal epilepsy [25]. In another experimental study with a small sample size, there was no difference found in the acceptability of BRV in both focal and generalized onset seizures, implying that BRV may be effective in both types of seizure patients [26]. Similarly, another study based on findings of two double-blind Phase III trials, investigated the efficiency, acceptability, and safety of concomitant BRV in patients with Unverricht-Lundborg disease (ULD) [27], which is an infrequent, progressive epilepsy that is described by generalized onset epileptic seizures and severe stimulus-sensitive myoclonus [28]. These trials showed an insignificant effect of BRV on action myoclonus. BRV was, however, generally well accepted by ULD patients [28].

Epilepsy treatment aims to control seizures adequately and enhance the quality of life with no adverse consequences of medication. Nonetheless, adverse consequences owing to the use of ASMs arise commonly and are the major cause of restricting doses recommended to achieve adequate seizure control [29]. Compromised treatment adherence may also be due to adverse consequences. Likewise, another study revealed that a significant fraction of epileptic patients stop their medication owing to adverse outcomes [30]. Previous studies reported that the negative effects of ASMs significantly impair the quality of life in epileptic patients [31,32].

There is no data available regarding the efficacy of BRV in patients with epilepsy in a real-world setting in the Pakistani population. Therefore, this real-world evidence was aimed at assessing the efficacy and safety of Brivera (referred to as BRV), manufactured by Helix Pharma Pvt Ltd., Sindh, Pakistan, monotherapy in patients with epilepsy in the Pakistani population.

## Materials and methods

This was a prospective, observational, multicenter study that was conducted in Pakistan at multiple sites (Karachi, Lahore, Islamabad, Faisalabad, Sialkot, Multan, Rahim Yar Khan, Peshawar, and Rawalpindi) from February 2022 to December 2022, by using a non-probability consecutive sampling technique. The ethical approval was taken from the Institutional Review Board, King Edward Medical University, Pakistan (342/RC/KEMU, dated February 15, 2022). The duration of the treatment was about three months after taking approval. The research sample was drawn from various private neurology clinics. The study population consisted of 368 naive adult patients of both genders with diagnosed epilepsy who were over the age of 18 and had focal seizures with or without secondary generalization and were willing to participate, while patients under the age of 18, pregnant or lactating women, those who were allergic to Brivera, and patients with a history of kidney or liver disease, depression or suicidal thoughts, and alcoholism were excluded.

Demographic characteristics, clinical history, type of seizures, and epilepsy of all patients were recorded. According to the recommendation of the physician, BRV monotherapy of dosage 25, 50, and 100 BD was prescribed to the newly diagnosed epileptic patients and they were followed up to three months. The clinical considerations determining dosage use for BRV monotherapy in newly diagnosed epileptic patients involve several factors. These considerations are crucial in tailoring the dosage to the individual patient's needs while optimizing therapeutic outcomes. Some key factors to consider include seizure control, patient characteristics, tolerability, adverse effects, and drug interactions. During BRV therapy, improvement in seizure episodes, associated side effects, and drug resistance at baseline and follow-up visits on the 14th and 90th days were documented. Outcomes included ≥ 50% reduction from baseline in seizure frequency, seizure freedom (no seizures within three months before the time point), continuous seizure freedom (no seizures from baseline), BRV discontinuation, and treatment-emergent adverse events (TEAEs) at three months. Patients with missing data after BRV discontinuation were considered non-responders/not seizure-free. The safety of the drug was observed, which consisted of assessing and recording all adverse effects associated with BRV therapy.

All the observed data was analyzed using IBM SPSS Statistics for Windows, Version 20.0 (Released 2011; IBM Corp., Armonk, New York, United States). Categorical variables were documented as frequencies and percentages. Monthly seizure episodes were presented as mean±SD. Chi-square test was applied to find out the association between seizure episodes, prescribed drugs, and follow-up visits. Moreover, paired t-test was applied between means of seizure episodes at baseline and follow-up visits. Prior to applying the paired t-test, we conducted a normality assumption check using the Shapiro-Wilk test. The results indicated that the differences between paired observations were approximately normally distributed (p > 0.05).A sub-group analysis was conducted to explore the potential dose-response relationship within each dosage group (25 mg, 50 mg, and 100 mg) for BRV. Descriptive statistics revealed varying levels of treatment compliance at the 90th day across the dosage groups, suggesting a potential trend. To further substantiate this observation, we performed formal statistical tests for trend (Cochran-Armitage test), which indicated a significant dose-response relationship (p-value < 0.05). A p-value of less than 0.05 was considered to be statistically significant.

## Results

A total of 368 epileptic patients with a mean age was 35.11±17.11 years were included in the study, of whom 187 (50.81%) were male and 181 (49.18%) were female. A total of 115 (31.3%) patients received a dosage of 25 mg of BRV, 153 (41.6%) patients were administered 50 mg, and 100 (27.1%) were prescribed a dosage of 100 mg. Concerning BRV compliance analysis, most of the patients (n=316; 85.86%) exhibited good treatment compliance on the 90th day; 52 (14.14%) patients were lost to follow due to non-improvement of condition, worsening of illness, or some other economic factors.

**Figure 1:**
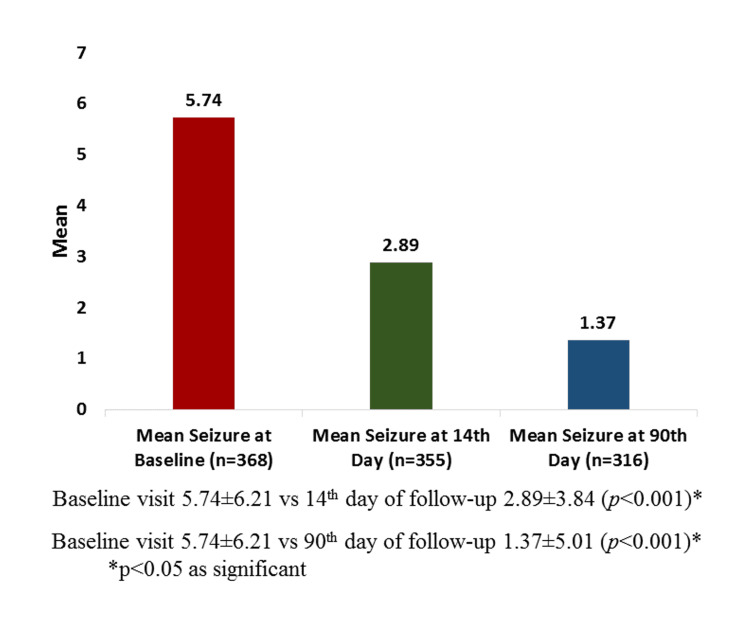
Mean of seizure episodes at baseline and follow-up visits.

The mean number of seizures at the baseline visit was 5.74±6.21. The mean number of seizure episodes on the 14th day of follow-up for 355 patients was 2.89±3.84. The mean number of seizure episodes on the 90th day of follow-up for 316 patients was 1.37±5.01. A comparison of baseline mean seizure per month with follow-up visits revealed that the mean reduction in seizure was 2.89±3.84 on the 14th-day visit from a baseline of 5.74±6.21 in 355 patients, reflecting a significant reduction in seizures (p<0.001), while the mean reduction in seizure was 1.37±5.01 on the 90th-day visit from the baseline of 5.74±6.21 in 316 patients, showing a significant reduction in seizures (p<0.001), as shown in Figure 1.

Overall, a more than 50% reduction in seizure episodes was achieved in 178 (56.3%) patients on day 90, and less than 50% reduction in seizure episodes was achieved by 95 (26.8%) patients on Day 14, with a highly significant association between them (p<0.001) as shown in Table 1.

**Table 1:** Comparison of seizure episodes on day 14 vs. day 90 Data given as n (%)

Seizure Episodes on Day 14(n=355)	Present	260 (73.2%)
	Absent	95 (26.8%)
Seizure Episodes on Day 90 (n=316)	Present	138 (43.7%)
	Absent	178 (56.3%)

**Figure 2:**
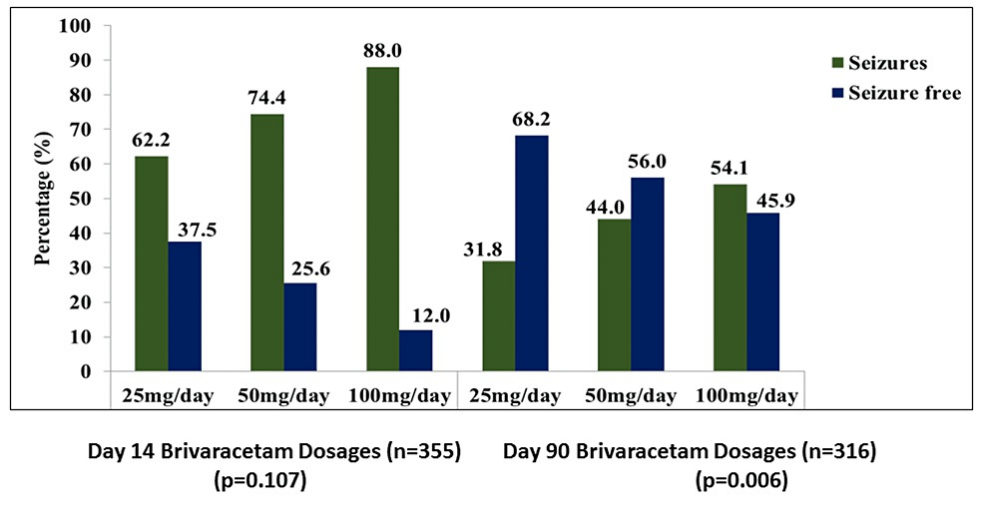
Brivaracetam dose-response analysis with or without seizures at follow-up visits

At day 14, BRV dose-response analysis of 355 patients showed a significant association between BRV dosages of 25, 50, and 100 mg/day and seizure-free and seizure episodes in patients, (p=0.107). On the 90-day visit of 316 patients, 32 (31.8%), 82 (44.0%), and 16 (54.1%) patients receiving BRV dosages of 25, 50, and 100 mg/day, respectively, had seizures. Additionally, at BRV dosages of 25, 50, and 100 mg/day, 68 (68.2%), 104 (56.0%), and 14 (45.9%) patients were seizure-free, respectively, with a significant association between them (p=0.006) as shown in Figure 2.

**Figure 3:**
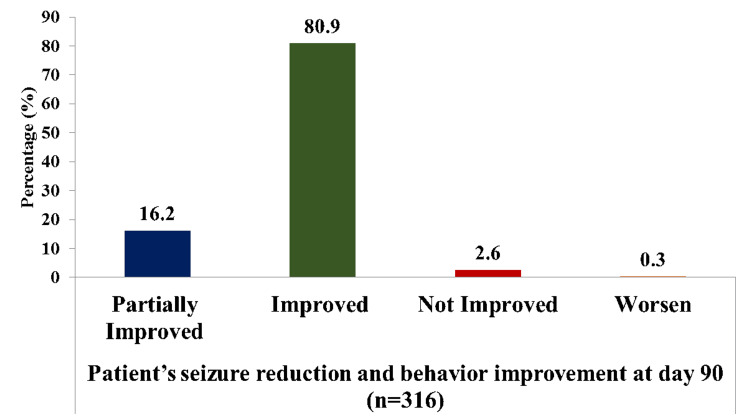
Improvement in patient’s overall condition (according to investigators’ subjective assessment) on the 90th day after BRV therapy Partially Improved: refers to a patient who, at the 90th day of assessment, experiences some degree of improvement in their overall condition, specifically in seizure frequency

On day 90, out of 316 patients 256 (80.9%) patients improved their overall condition (seizure) and 51 (16.2%) partially improved (Figure 3).

**Figure 4:**
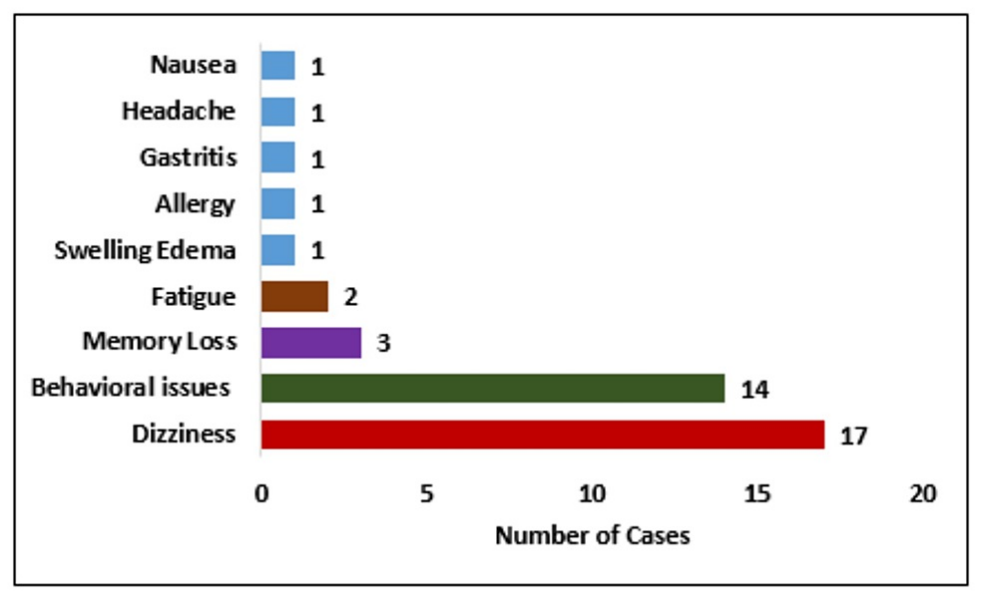
Frequency of patients with adverse events during brivaracetam (BRV) therapy

Among 316 patients, only 41 (4.4%) of all BRV-treated patients experienced adverse events. Of these 41 patients, 17 (41.7%) patients reported dizziness and 14 (34.2%) reported behavioral issues as shown in Figure 4.

## Discussion

This study demonstrated the effects on seizure frequency after treatment with BRV in epileptic patients. Seizure freedom is one of the ultimate objectives of thThe current study, which indicated 41 (13%) patients erapy in patients with epilepsy. It is reported that reductions in seizure frequency that fall short of seizure freedom have little impact on the quality of life of patients with treatment-resistant epilepsy and the International League against Epilepsy promotes seizure freedom as a key study outcome. Despite its significance, many trials do not publish the seizure freedom result, and its definition exhibits significant variation [33].

The primary option for treating epilepsy that is widely accepted is monotherapy. However, the patient is offered a trial of combination medication if seizures continue even after being treated with the highest acceptable dose of a single ASM [34]. Many studies reported that monotherapy is effective in treating epileptic seizure episodes; 82.2% of patients in the study by Ayalew and Muche, and 80.35% of patients with epilepsy in the study by Birru et al. receiving monotherapy significantly reduced seizure episodes [35,36]. According to studies by Rishe et al. and Getnet et al., conducted in Ethiopia, monotherapy was administered to 78.6% and 76.7% of epileptic patients, respectively [37,38]. The findings of these studies were similar to the findings of the present study and showed that the majority of the patients (56.3%) who were seizure-free were getting monotherapy, which significantly (p< 0.001) decreased seizure episodes after three months of therapy.

As far as the patient’s seizure improvement is concerned, in a study by Menzler et al., it was observed that 44% of patients had a reduced seizure episode, 38% had no change, 18% of patients reported an increased seizure episode, and 17% of patients were seizure-free after receiving BRV. Most of the patients (n=19; 63%) improved their epilepsy by administration of BRV monotherapy [39]. The present study did not support the above research findings and revealed that the majority of the patients (n=178; 56.3%)were seizure-free at day 90 after initiation of BRV and reported none of the treated patients increased seizure episodes.

There is limited evidence-based data reported that support the reduction in long-term persistent seizure frequency with BRV treatment. In randomized controlled trials with a 12-week treatment term, a similar methodology has been used to analyze the time course of add-on BRV effectiveness and measure 50% and 100% response [40,41]. In a study by Klein et al., the 100% response rates for BRV 100 mg/day and 200 mg/day treatment were 5.1% and 4.0%, respectively, on day 1 of therapy in the pooled efficacy-evaluating group of 1160 patients and remained mostly constant by day 84 [40]. According to another study, patients using BRV at doses of 50 mg/day, 100 mg/day, and 200 mg/day attained 50% seizure-free response on day 1 by 15.5%, 18.1%, and 19.4%, respectively, while similar values on day 84 were 34.8%, 35.8%, and 35.2%, respectively [42]. The current study differed from the aforementioned studies in that it showed 68.2%, 56%, and 45.9% seizure-free patients on day 90 on doses of 25 mg/day, 50 mg/day, and 100 mg/day BRV. Additionally, the current study recommended BRV doses of 25 mg/day, 50 mg/day, and 100 mg/day, which was also inconsistent with the previously mentioned studies.

The current study indicated 41 (13%) patients experienced adverse effects. This is the least among the studies reviewed by us but is similar to the results of the study by Ayalew and Muche, which stated that 15% of patients reported unfavorable effects from ASM treatment [35], and with the 17.6% adverse effects reported in the study conducted by Birru et al. in 2014 [36]. However, in a study conducted in northwest Ethiopia, 25.8% of epileptic patients who were on ASM treatment had adverse events [37], which was quite higher than the current study.

Ayalew and Muche reported various types of adverse events such as exhaustion, GI disruption, and sedation/depression [35]. Headache was the most common adverse effect documented in another study [38]. Birru et al. observed that hypersomnia was the most common side effect of ASM treatment [36]. The present study was inconsistent with the aforementioned studies and found that the most frequent side effects of ASMs were dizziness, which was reported by 17 (41.7%) patients, followed by behavioral problems, which were reported by 14 (34.2%) patients.

While the current study presents valuable insights into the effectiveness and tolerability of BRV in a real-world clinical setting, we acknowledge certain limitations inherent to its observational nature and non-random sampling method. The absence of randomization introduces potential selection bias, and the non-methodical data gathering, irregular follow-up times, and co-medication alterations reflect the challenges encountered in routine clinical practice. Importantly, our inclusive criteria, incorporating all patients receiving BRV with at least a three-month follow-up, renders our study highly applicable to typical clinical scenarios in Pakistan. We believe that by transparently acknowledging and discussing these limitations, our study contributes meaningfully to the existing literature on BRV, offering practical insights for clinicians and researchers alike.

## Conclusions

The outcomes of this study imply that BRV monotherapy is an effective and well-tolerated option for treating individuals with epilepsy in the Pakistani population. The remarkable decrease in seizure episodes, especially the encouraging early response observed by Day 14, underscores its potential to enhance the quality of life for those grappling with epilepsy. Moreover, the favorable safety profile, marked by a relatively low occurrence of adverse events, further bolsters the positive prospects of BRV as a credible treatment choice. These findings contribute to our comprehension of the role of BRV in the management of epilepsy and provide valuable insights for healthcare professionals and patients in Pakistan, as well as potentially in regions with similar characteristics. Ongoing research and extended follow-up studies hold the promise of unveiling more insights into the sustained effectiveness and safety of BRV in the realm of epilepsy treatment.
